# Ginsenoside Rh7 affects β-catenin nuclear translocation by inhibiting *SHCBP1* expression, thereby inhibiting epithelial-mesenchymal transition in gastric cancer cells

**DOI:** 10.7150/ijms.112622

**Published:** 2025-06-23

**Authors:** Xiaohong Zhang, Yanqing Mo, Li Feng

**Affiliations:** 1 Endoscopy Center, Minhang Hospital, Fudan University, No. 170 Xinsong Road, Shanghai, 201199, China.; 2 Shanghai Minhang District Fudan Medical Education and Research Collaborative Development Institute, China.

**Keywords:** ginsenoside Rh7, gastric cancer, *SHCBP1*, β-catenin, epithelial-mesenchymal transition

## Abstract

**Background:** Ginsenoside Rh7 is a bioactive compound with anticancer properties. This investigation was conducted to analyze the anticancer effects of ginsenoside Rh7 and its underlying molecular mechanisms in gastric cancer (GC) cells.

**Methods:** The key gene module associated with GC was identified through weighted gene co-expression network analysis (WGCNA) of the GSE118897 dataset. Differentially expressed genes (DEGs) were examined in The Cancer Genome Atlas-Stomach Adenocarcinoma (TCGA-STAD) and the GSE118897 datasets. The central genes of this study were subsequently identified by intersection analysis and protein-protein interaction (PPI) network. Transcriptome sequencing evaluated the changes in *SHCBP1* expression in GC cells treated with Rh7. Immunoprecipitation (IP) was employed to analyze the relationship between β-catenin and *SHCBP1*. Functional assays, including Transwell, cell counting kit-8 (CCK-8), colony assays, and *in vivo* tumor models, evaluated the effects of Rh7 and *SHCBP1* on GC cell behaviors.

**Results:** *SHCBP1* was upregulated in tumor samples in GSE118897 and TCGA-STAD. Ginsenoside Rh7 inhibited GC cell invasion, migration, and proliferation dose-dependently by downregulating *SHCBP1* expression. Transcriptome analysis confirmed Rh7-mediated *SHCBP1* inhibition. Rh7 promoted β-catenin nuclear translocation by reducing *SHCBP1* expression. Rescue experiments demonstrated that the overexpression of *SHCBP1* partially counterbalanced the impacts of Rh7 on epithelial-mesenchymal transition (EMT) regulation and GC cell growth *in vitro* and *in vivo*.

**Conclusion:** Ginsenoside Rh7 suppresses GC progression by regulating SHCBP1-mediated β-catenin nuclear translocation, thereby inhibiting EMT, proliferation, migration, and invasion. This highlights its potential as a GC therapeutic drug and deserves further study of its mechanism of action.

## 1. Introduction

Gastric cancer (GC) is a prevalent malignancy worldwide, the fourth most frequent cause of cancer-related fatalities worldwide and the fifth most prevalent cancer^1^. Studies predict that the global burden of GC will increase by 62% by 2040^2^. Helicobacter pylori (H. pylori) infection is known to be the result of nearly 90% of distal GC cases, and other risk factors involve nitrate-rich foods, a high-salt diet, alcohol consumption, smoking, and genetic predisposition^3^, ^4^. Currently, the most common therapies include surgery, chemotherapy, and targeted therapy^5^, ^6^. Docetaxel, oxaliplatin, and 5-fluorouracil (5-FU) are common chemotherapy medications^7^. However, for advanced gastric cancer, clinical outcomes remain unsatisfactory, and new treatment strategies are needed. Traditional Chinese medicine (TCM) has recently attracted attention as a complementary or alternative approach to GC treatment^8^. Notably, ginsenosides, a bioactive compound from traditional Chinese medicine, exhibit promising anticancer effects by regulating autophagy, apoptosis, proliferation, migration, and angiogenesis in various gastrointestinal cancers.

Ginsenosides are sterol compounds isolated and extracted from ginseng, which have various biological activities, such as anti-inflammatory, antitumor, immunomodulatory, and antioxidant impacts^9^. Based on their chemical structures, ginsenosides can be divided into protopanaxadiol, protopanaxatriol and oleanolic acid types^10^. Increasing evidence supports their potential in cancer treatment, among which specific ginsenosides such as Rh4 and Rd exhibit significant anti-GC effects. For example, the SIX1-TGF-β/Smad2/3 axis is limited by ginsenoside Rh4, which inhibits GC cells from migrating and invading^11^. Ginsenoside Rd induces GC cell apoptosis and cycle arrest by upregulating Bax/Bcl-2 ratio, Caspase-9, and Caspase-3^12^. Despite these advances, the therapeutic potential of ginsenoside Rh7, a rare protopanaxatriol-type saponin, remains underexplored. Recent studies have shown that Rh7 suppresses the progression of non-small cell lung cancer (NSCLC) via downregulating ILF3-AS1 expression^13^. However, its anti-GC mechanism and clinical relevance have not been cleared. Therefore, our investigation focused on uncovering the mechanism of action of ginsenoside Rh7 in GC and further analyzing its therapeutic potential in tumors.

SHC binding protein 1 (SHCBP1) is a protein that interacts with SH2 domain-containing signaling molecules such as Shc and is crucial for controlling cell growth, differentiation, and apoptosis^14^. Recent studies have highlighted the important function of* SHCBP1* in tumor progression. For example, Lin Y and Cai H provided a comprehensive overview of *SHCBP1* expression, its biological functions, and its potential clinical significance in several cancers^15^. These included that *SHCBP1* knockdown prevented the cell proliferation of hepatocellular carcinoma (HCC) and induced G0/G1 arrest by downregulating p-ERK1/2 and cyclin D1 expression, emphasizing its involvement in MEK/ERK signaling. *SHCBP1* enhances metastasis and invasion of pancreatic cancer cells through Notch1 signaling and epithelial-mesenchymal transition (EMT) processes. *SHCBP1* also was found implicated in tumor growth, metastasis, and invasion of bladder cancer, prostate cancer, and breast cancer^16^, ^17^. In addition, another study revealed that *SHCBP1* was markedly expressed in GC tissues and was linked to cancer progression and a bad prognosis for patients^18^. Knockdown of *SHCBP1* effectively suppressed GC cell migration, proliferation, invasion, and EMT, demonstrating its potential as a GC treatment target and prognostic indicator. These examples confirm the key role played by* SHCBP1* in various cancer types and emphasize its importance in the study of cancer as a molecular target.

Although the biological function of *SHCBP1* and its effects on several kinds of tumors have been thoroughly investigated, its role in GC remains underexplored. Furthermore, ginsenosides have demonstrated promise anticancer activities, although the exact molecular processes of ginsenoside Rh7 in GC, notably its interaction with* SHCBP1*, have yet to be assessed. Consequently, the goal of this research was to explore the antitumor impacts of ginsenoside Rh7 on gastric cancer cells by focusing on its regulation of *SHCBP1* expression, β-catenin translocation, and EMT regulation to provide new insights into the GC targeted treatment.

## 2. Materials and methods

### 2.1 Data source

The GSE118897 dataset was obtained from the Gene Expression Omnibus (GEO, https://www.ncbi.nlm.nih.gov/gds/), which contains 10 GC tissue samples and 10 normal gastric mucosa tissue samples. Additionally, 375 stomach adenocarcinoma (STAD) samples and 32 adjacent non-tumorous samples were obtained through the Sangerbox platform (http://vip.sangerbox.com/home.html) retrieved from The Cancer Genome Atlas (TCGA) dataset.

### 2.2 Identification of GC-related gene modules through gene co-expression network

To determine which gene modules are linked to GC, a weighted gene co-expression network analysis (WGCNA) was conducted on the GSE118897 dataset. The gene expression data, which encompass the entire transcriptome of the dataset, were transformed into a scale-free co-expression network. The optimal soft threshold power for network construction was identified as 28, based on the scale-free topological fit index (R^2^ > 0.85). Subsequently, hierarchical clustering dendrograms were generated, and dynamic tree cutting was employed to identify distinct gene modules. These modules were identified based on their co-expression patterns and assigned unique colours for visualisation. Module feature genes were then associated with phenotypic features (tumour and normal samples). Finally, heat maps of feature gene adjacency and module-feature correlation were constructed to identify modules significantly associated with GC for the subsequent stage of analysis.

### 2.3 Identification and intersection analysis of GC-related differentially expressed genes (DEGs)

Differential expression analysis was executed on the GSE118897 and TCGA-STAD GC datasets utilizing the "Limma" package in R (version 3.42.2). For both datasets, genes with a fold change (FC) < 0.5 were downregulated, while those with an FC > 2 were deemed upregulated. The statistical significance of DEGs was determined by applying a significance threshold of *P* < 0.05. Following this, Venn diagram analysis was performed by the Venn online graph tool (https://bioinformatics.psb.ugent.be/webtools/Venn/) to determine overlapping upregulated and downregulated DEGs between the GSE118897 dataset, TCGA-STAD dataset, and the turquoise module. This overlap highlighted potential key genes associated with GC progression.

### 2.4 Protein-protein interaction (PPI) network analysis and candidate gene identification

Overlapping genes were analyzed using the Search Tool for the Retrieval of Interacting Genes (STRING) database (https://string-db.org/) to establish a PPI network. The cytoHubba plugin of Cytoscape software was employed to rank the top 10 genes based on two algorithms: MNC (Maximum Neighborhood Component) and DMNC (Density of Maximum Neighborhood Component). The Venn online graph tool (https://bioinformatics.psb.ugent.be/webtools/Venn/) was used to determine candidate genes by performing a topological analysis of the top ten genes from both algorithms. Furthermore, gene expression levels in tumor and normal samples were analyzed and visualized as boxplots using Sangerbox (3.0, http://vip.sangerbox.com/home.html) to assess the significance of candidate gene expression.

### 2.5 RNA extraction and sequencing of SGC-7901 cells treated with ginsenoside Rh7

SGC-7901 cells were exposed to DMSO or 50 μM ginsenoside Rh7 for 24 h, and total RNA was extracted. Three biological replicates were performed for each treatment group to ensure reproducibility. In accordance with the manufacturer's instructions, TRIzol reagent (Tiangen, Beijing, China) was employed for RNA extraction. The purity and concentration of the extracted RNA were detected by a NanoDrop 2000 spectrophotometer (Thermo Scientific, Shanghai, China), while RNA integrity was detected using an Agilent 2100 Bioanalyzer (Agilent, Shanghai, China). By the manufacturer's instructions, RNA sequencing libraries were produced by the VAHTS Universal V6 RNA-seq Library Prep Kit. Sequencing was occurred on the Illumina NovaSeq 6000 platform, which provides high-throughput and high-quality sequencing data. Shanghai Meijie Biopharmaceutical Technology Co., Ltd. performed all library preparation and sequencing procedures. The obtained sequencing data were subjected to differential expression analysis, and the screening conditions were set as the criteria for down-regulated DEGs (FC<0.5) and up-regulated DEGs (FC>2), and both satisfied P<0.05. Then, the expression of *SHCBP1* in the DMSO control and Rh7 treatment groups was further detected.

### 2.6 Cell cultured and reagents

This study utilized human gastric cancer cell lines (AGS, MGC803, SGC-7901, and SNU-1) and human normal gastric epithelial cells (GES-1), which were sourced from BioVector NTCC (Beijing, China). These cells were maintained in RPMI-1640 medium added 10% fetal bovine serum (FBS) and incubated with 5% CO_2_ at 37°C in a humidified atmosphere. Additionally, ginsenosides RC, Rh7, Rh1, and Rh3, along with the organic solvent dimethyl sulfoxide (DMSO), were acquired from Shanghai Yuanye Bio-Technology Co., Ltd.

### 2.7 Ginsenoside treatment protocol

Ginsenosides (RC, Rh7, Rh1, or Rh3) were first dissolved in DMSO to create a stock solution with a concentration significantly higher than the required working concentrations. To reach a final concentration of 100 μM for each ginsenoside, the stock solution was then diluted in full cell culture medium. For the specific evaluation of ginsenoside Rh7, the stock solution was diluted in complete cell culture media to achieve final doses of 0 μM (control), 1 μM, 5 μM, 10 μM, 50 μM, and 100 μM. To minimize cytotoxicity, the concentration of DMSO in the media was maintained below 0.1%. The ginsenoside-containing medium was applied to the cells, and they were then incubated for 48 hours with 5% CO_2_ at 37°C. For the control group, media containing equivalent concentrations of DMSO without ginsenoside was used to ensure consistency in solvent concentration across all treatment groups.

### 2.8 Cell transfection

SGC-7901 and AGS cells were planted in 24-well plates at a density of 2 × 10^5^ cells per well and cultured overnight in complete growth medium to reach approximately 70-80% confluence. Lipofectamine 2000 reagent (Invitrogen) was employed for transfection following the manufacturer's instructions. Specifically, *SHCBP1* overexpression plasmids (over-*SHCBP1*) or empty vector controls were transfected into SGC-7901 and AGS cells. All experiments were conducted using a standardized transfection protocol. For further examination, cells were extracted 48 hours after transfection.

### 2.9 Cell proliferation assay

To determine the proliferation of cell, the Cell Counting Kit-8 (CCK-8) test was applied. Briefly, SGC-7901 and AGS cells were planted at a density of 5 × 10³ cells per well in 96-well plates. For the initial experiment, cells were exposed to 100 μM of various ginsenosides, including RC, Rh7, Rh1, and Rh3. After treatment for 24 h, each well received 10 μL of CCK-8 reagent (Beyotime, Shanghai, China), and the plates were then cultured at 37°C for 2 h. In a separate set of experiments, SGC-7901 and AGS cells were treated with 50 μM ginsenoside Rh7 for 1, 2, 3, and 4 days. At the specified times, each well was treated with 10 μL of CCK-8 reagent and incubated for 2 hours. The absorbance at 450 nm was employed to detect cell viability, and a proliferation curve was generated. Control wells were treated with an equivalent volume of DMSO.

### 2.10 Half-maximal inhibitory concentration (IC_50_) assay

The half-maximal inhibitory concentration (IC_50_) of ginsenosides in SGC-7901 and AGS cells was established by the CCK-8 assay. Cells were exposed to ginsenoside Rh7 at several doses (0, 1, 5, 10, 50, and 100 μM) for 24 h. The experimental procedure followed the same protocol as described in the CCK-8 assay. A microplate reader was applied to identify absorbance at 450 nm to evaluate cell proliferation. GraphPad Prism software (GraphPad Software, San Diego, CA, USA) was implemented to calculate the IC_50_ values.

### 2.11 Transwell migration and invasion assays

Cell invasion and migration were assessed utilizing Transwell chambers (8 μm pore size, Corning, NY, USA). To conduct the migration assay, SGC-7901 and AGS cells were planted at a density of 1 × 10^5^ cells per well into the upper chamber (without any coating), suspended in serum-free RPMI-1640 medium. The cells were treated with DMSO, Ginsenoside Rh7 (50 μM), or Ginsenoside Rh7 (50 μM) combined with overexpression of *SHCBP1*. The top chambers were pre-coated with Matrigel (BD Biosciences, San Jose, CA, USA) for the invasion investigation, and the Matrigel was allowed to harden for 30 minutes by incubating them at 37°C. Afterward, cells were seeded in the upper chambers in serum-free medium at a density of 1 × 10^5^ cells per well, while 10% FBS was present in the medium in the lower chambers. A cotton swab was employed to gently remove any non-invading or non-migrating cells from the membrane's upper surface after a 24-hour period. For 20 minutes at room temperature, the migrating or infiltrated cells on the bottom surface were preserved with 4% paraformaldehyde. Subsequently, 1 μg/ml 4′,6-diamidino-2-phenylindole (DAPI) was applied to label the cells for 10 minutes to visualize the cell nuclei. Five randomly chosen fields per chamber were utilized for counting the number of migratory or invading cells using a fluorescence microscope to facilitate comparison between different treatment groups.

### 2.12 Colony formation assay

For the assay of colony formation, 200 cells from each treatment group (SGC-7901 and AGS) were seeded into each well of a 6-well plate and cultured in RPMI-1640 medium with 10% FBS added for 10 days. After the incubation period, the cells were preserved with 4% paraformaldehyde after being washed with phosphate-buffered saline (PBS) for 15 minutes at room temperature. After fixation, the cells were washed again with PBS to remove excess fixative. Subsequently, colonies were stained using nitro blue tetrazolium chloride solution. A microscope (Olympus, Tokyo, Japan) was employed to take pictures of the colonies, and the amount of colony formation was manually counted for quantification.

### 2.13 RNA Extraction and Quantitative Real-Time PCR (qRT-PCR)

Total RNA was extracted from SGC-7901 and AGS cells utilizing TRIzol reagent (Tiangen, Beijing, China) following the manufacturer's instructions. With the PrimeScript RT Reagent Kit (Takara, Dalian, China), 1 μg of total RNA was reverse-transcribed into complementary DNA (cDNA). qRT-PCR was cconducted with SYBR Green Master Mix (Roche, Basel, Switzerland) on a QuantStudio 3 Real-Time PCR System (Thermo Fisher Scientific, Waltham, MA, USA). The 2^-ΔΔCt^ method was applied to quantify relative gene expression levels, with *GAPDH* serving as the internal control^19^. The primer sequences employed for qRT-PCR were as follows: *SHCBP1*: 5'-TTGAAATGGCTGACGGGTCG-3', 5'-GCCTTGCAGTCAGCTGGTTT-3', *N-Cadherin*: 5'-GTGCATGAAGGACAGCCTCT-3', 5'-GGGTCTCTTTGTCTTGGGCA-3', *E-Cadherin*: 5'-ATGCTGATGCCCCCAATACC-3', 5'-GAACAGCTGTGAGGATGCCA-3', *Vimentin*: 5'-TGGACCAGCTAACCAACGAC-3', 5'-GCCAGAGACGCATTGTCAAC-3', *GAPDH*: 5'-GACAGTCAGCCGCATCTTCT-3', 5'-GCGCCCAATACGACCAAATC-3'.

### 2.14 Western blotting (WB)

Protease and phosphatase inhibitors (Thermo Fisher Scientific, Waltham, MA, USA) were added to RIPA lysis buffer (Beyotime, Shanghai, China) to create cell protein extracts. SDS-PAGE was employed to separate equal quantities of protein (30 μg), which were then deposited onto PVDF membranes (Millipore, Billerica, MA, USA). The membranes were then blocked with 5% non-fat milk in TBST for 1 h and incubated with primary antibodies overnight at 4°C: SHCBP1 (1:1000, ab184467), β-catenin (1:5000, ab32572), P84 (1:10000, ab131268), E-cadherin (1:1000, ab40772), N-cadherin (1:1000, ab245117), Vimentin (1:1000, ab92547), and GAPDH (1:20000, ab128915), all of which were purchased from Abcam (Shanghai, China). After washing with TBST, the membranes were treated with HRP-conjugated secondary antibodies (1:3000, Goat Anti-Rabbit IgG H&L, HRP, ab6721, Abcam) at room temperature for 1 h. Protein bands were visualized utilizing an ECL detection system (Thermo Fisher Scientific).

### 2.15 Immunoprecipitation (IP)

To investigate the interaction between *SHCBP1* and β-catenin, IP was performed. IP lysis buffer (Beyotime, Shanghai, China) was employed to lyse the cells, to put it briefly. For total and nuclear protein extraction, lysates were prepared separately to analyze both total and nuclear fractions. To immunoprecipitate SHCBP1-associated proteins, cell lysates were treated with an anti-SHCBP1 antibody at 4°C overnight. Similarly, to examine β-catenin-associated proteins, lysates were incubated with an anti-β-catenin antibody under the same conditions. The protein-antibody complexes were then captured by incubating with protein A/G agarose beads (Thermo Fisher Scientific, Waltham, MA, USA) for 2 h. After extensive washing, the immunoprecipitates were eluted and analyzed to Western blot to isolate and examine the targeted protein.

### 2.16 Animal xenograft model to assess the effects of ginsenoside Rh7 and SHCBP1 overexpression on GC tumor development

In this study, male mice aged 6-8 weeks were used for xenograft experiments to evaluate the effects of ginsenoside Rh7 and *SHCBP1* overexpression on GC tumor development. Four mice were used in each group, for a total of four groups. All animal experiments were approved by the Ethics Committee of the Laboratory Animal Science Department of Fudan University (Approval No.: 2024-MHYY-523) and followed the guidelines of the Guide for the Care and Use of Laboratory Animals. GC cells (SGC-7901) were suspended in phosphate-buffered saline (PBS), and each mouse received a subcutaneous injection of 1 × 10^6^ cells in 100 μL PBS into the right side. Four groups of mice were randomly selected: 1 DMSO control group, 2 ginsenoside Rh7 (50 μM) treatment group, 3 ginsenoside Rh7 (50 μM) + vehicle group, and 4 ginsenoside Rh7 (50 μM) +* SHCBP1* overexpression group. Drug treatment began after tumor formation, and the body weight and tumor size of the mice were measured every three days. At the end of the experiment, the mice were killed by carbon dioxide asphyxiation, and the tumors were removed and weighed.

### 2.17 Statistical analysis

We used R language to examine our data. The mean ± SD was conducted to represent all the data. The Student's t-test assessed group differences, while analysis of variance (ANOVA) with Tukey's post hoc test determined differences among multiple groups. The survival ratio and the difference among the groups were assessed by the KM method and log-rank test. The obvious statistical difference meant *P*< 0.05.

## 3. Results

### 3.1 Identification of key gene modules associated with GC in the GSE118897 dataset using WGCNA

**Figure 1:**
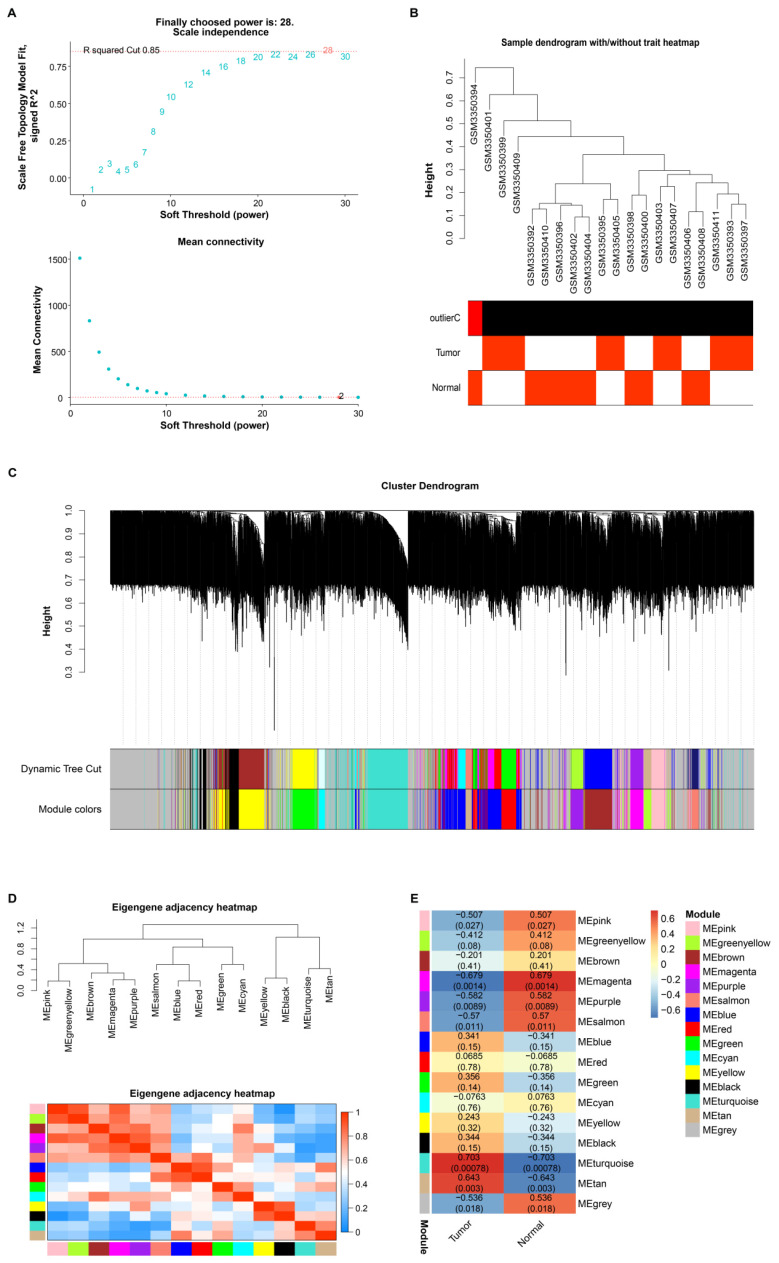
** WGCNA identification of key gene modules associated with GC in the GSE118897 dataset.** (A) Determination of the optimal soft-threshold power 28 to ensure a scale-free topology model fit. (B) Dendrogram of 20 samples from the GSE118897 dataset, showing the sample clustering with and without trait heatmaps. (C) Clustering dendrogram showing the division of genes into distinct modules based on their co-expression patterns, with each module represented by a unique color. (D) Heatmap of eigengene adjacency, visualizing the relationships between the 14 identified modules. (E) Correlation heatmap illustrating the relationships between different gene modules and the Normal and Tumor samples from the GSE42955 dataset.

As depicted in Figure 1A, the optimal soft-threshold power for ensuring a scale-free topology model was determined to be 28. Following the establishment of this threshold, we proceeded to analyze 20 samples from the GSE118897 dataset (Figure 1B). Through the application of the WGCNA method, genes were categorized into distinct modules based on their co-expression patterns, with each module being assigned a unique color (Figure 1C). To further elucidate the relationships between these identified modules, we conducted a detailed examination of the eigengene adjacency (Figure 1D). Notably, the turquoise module had a correlation coefficient of 0.703 with tumor samples of the GSE118897 dataset and a correlation coefficient of -0.703 with normal samples, indicating that the turquoise module was significantly associated with GC (Figure 1E).

### 3.2 Identification of hub gene through differential expression and PPI network analysis in GC

**Figure 2:**
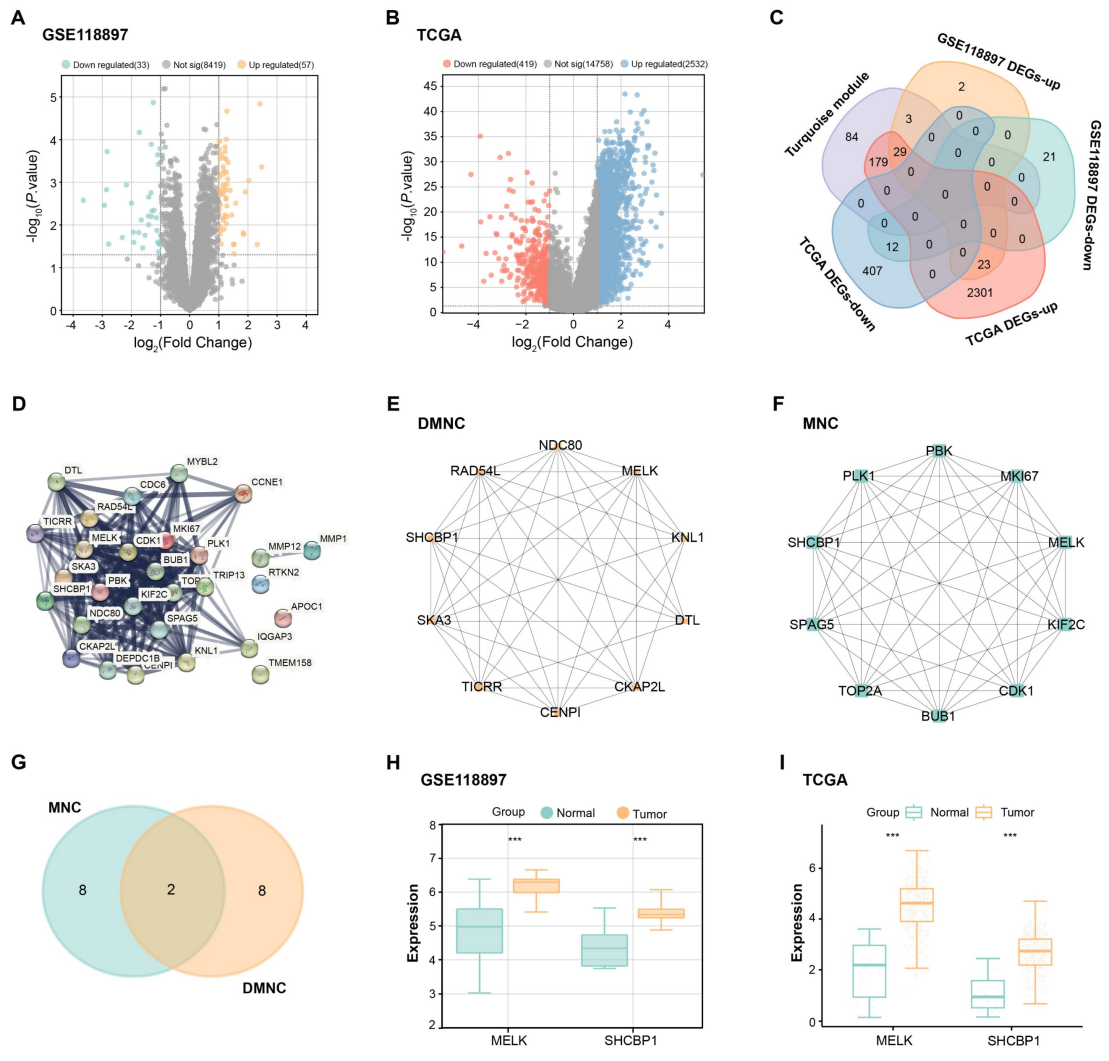
** Differential expression and PPI network analysis reveal hub genes in GC.** (A) Volcano plot illustrating the differential expression of genes in the GSE118897 dataset, with upregulated genes highlighted in orange and downregulated genes in cyan. (B) Volcano plot depicting DEGs in the TCGA-STAD dataset, with upregulated genes shown in blue and downregulated genes in red. (C) Venn diagram displaying the overlap of genes between the turquoise module and the DEGs from both the GSE118897 and TCGA-STAD datasets. (D) Protein-protein interaction (PPI) network analysis of the intersecting genes, comprising 26 nodes and 239 edges. (E-F) PPI network analysis of the intersecting genes based on the top 10 interacting genes identified by the DMNC (E) and MNC (F) algorithms, with 10 nodes and 41 edges for DMNC and 10 nodes and 45 edges for MNC. (G) Venn diagram illustrating the two overlapping genes identified by both the DMNC and MNC algorithms. (H-I) Expression levels of *SHCBP1* and *MELK* in both the GSE118897 and TCGA-STAD datasets. TCGA-STAD: The Cancer Genome Atlas Stomach Adenocarcinoma; DEGs, differentially expressed genes; PPI, protein-protein interaction; DMNC, density of maximum neighborhood component; MNC, maximum neighborhood component. ***P<0.001.

Differential expression analysis of the GSE118897 dataset and the TCGA-STAD dataset was performed using the R package. 33 downregulated and 57 upregulated DEGs were found from the GSE118897 dataset (Figure 2A). Analysis of the TCGA-STAD dataset showed 2,532 upregulated DEGs and 419 downregulated DEGs (Figure 2B). Subsequent topological analysis identified a total of 29 upregulated intersection genes from the turquoise module and the upregulated DEGs of the two datasets (Figure 2C). Further exploration of the interactions between these intersection genes resulted in a PPI network containing 26 nodes and 239 edges (Figure 2D). The DMNC and MNC algorithms highlighted the top 10 interacting genes, respectively (Figures 2E and 2F). From these genes, *MELK* and* SHCBP1* became the overlapping genes identified by the two algorithms (Figure 2G). The expression of *MELK* and *SHCBP1* was then evaluated in the GSE118897 and TCGA-STAD datasets, and the findings demonstrated that both genes were markedly overexpressed in tumor tissues (Figures 2H and 2I). It is important to note that since *SHCBP1* has been less documented in GC, we selected it as the hub gene in this study.

### 3.3 Ginsenoside Rh7 inhibits GC cell proliferation through dose-dependent mechanisms

**Figure 3:**
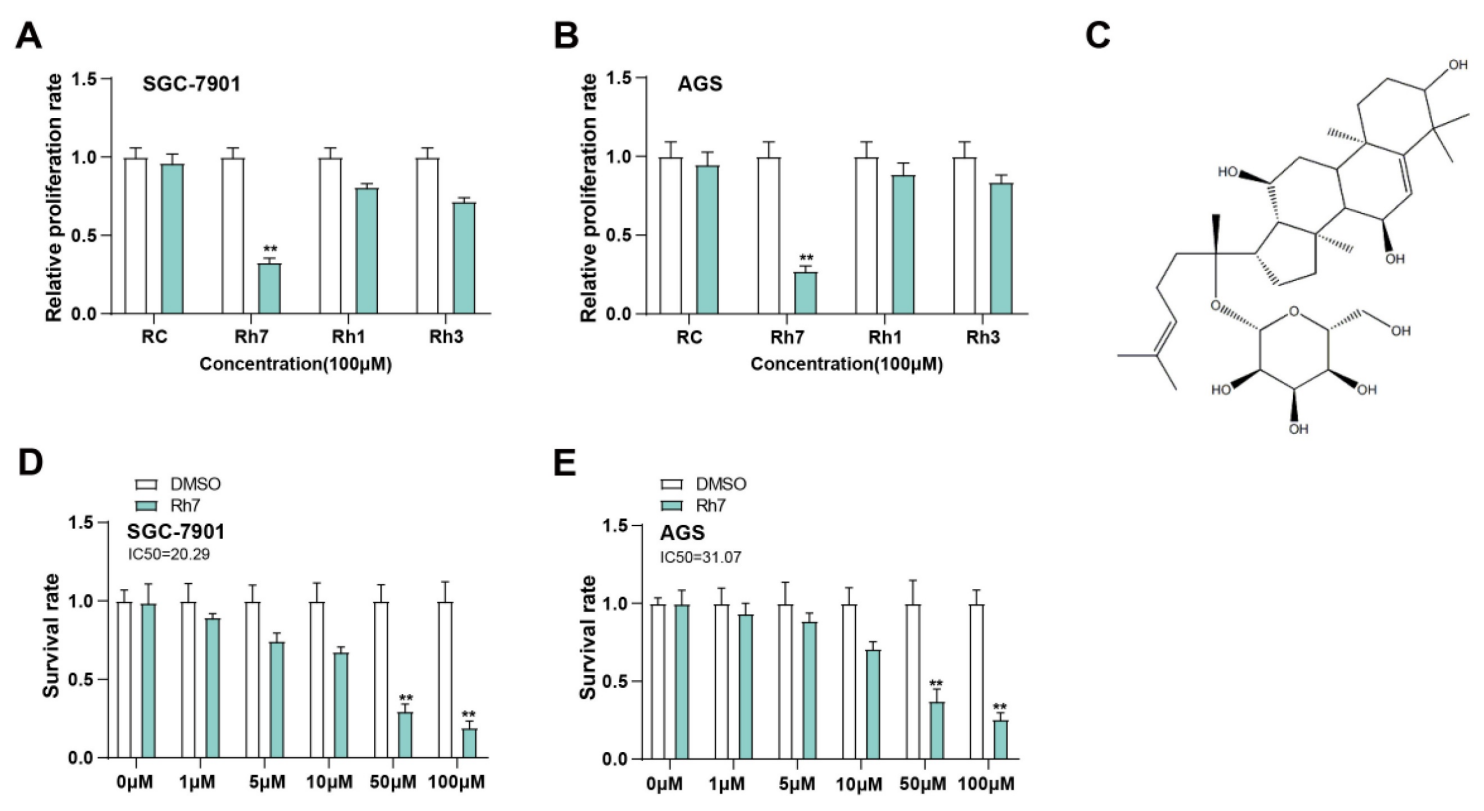
** Inhibitory effects of ginsenosides on GC cell proliferation.** (A and B) CCK-8 assay showing the effects of various ginsenosides (RC, Rh7, Rh1, and Rh3) at 100 μM on the proliferation of SGC-7901 and AGS cells. The x-axis represents different ginsenosides, and the y-axis represents the relative proliferation rate. (C) Chemical structure of ginsenoside Rh7. (D and E) IC_50_ values for ginsenoside Rh7 treatment at various concentrations (0, 1, 5, 10, 50, and 100 μM) in SGC-7901 and AGS cells. The x-axis represents different concentrations, and the y-axis represents the survival rate. GC, gastric cancer; CCK-8, cell counting kit-8; IC_50_, half-maximal inhibitory concentration; DMSO: Dimethyl sulfoxide. **P<0.01 vs. DMSO group.

To evaluate the antiproliferative impacts of different ginsenosides on GC cells, SGC-7901 and AGS cells were exposed to 100 μM RC, Rh7, Rh1, and Rh3 for 24 h (Figures 3A and 3B). The outcomes of the CCK-8 assay demonstrated that only Rh7 inhibited SGC-7901 and AGS cell proliferation. No significant inhibitory effect was observed after RC, Rh1, or Rh3 treatment, indicating that Rh7 has specific antiproliferative activity. The molecular formula of Rh7 is C_36_H_60_O_9_, and the chemical structure is shown in Figure 3. Next, GC cells were exposed to increasing doses of Rh7 (0, 1, 5, 10, 50, and 100 μM) to detect the cytotoxic effect of Rh7. As shown in Figures 3C and 3D, Rh7 exhibited a dose-dependent suppression of the viability of both cell lines. At 50 μM and 100 μM, the viability of SGC-7901 and AGS cells was significantly reduced to less than 50%. Specifically, the IC_50_ value of the SGC-7901 cell line was 20.29 μM, while that of the AGS cell line was 31.07 μM, indicating that Rh7 had a more significant inhibitory effect on the SGC-7901 cell line at a lower concentration. These outcomes suggest that Rh7 has a dose-dependent impact on GC cell survival and proliferation.

### 3.4 Transcriptome profiling and validation of SHCBP1 expression in GC cells after ginsenoside Rh7 treatment

This investigation applied transcriptome sequencing to analyze the gene expression changes in GC cells treated with ginsenoside Rh7. Differential expression analysis identified 96 upregulated DEGs and 112 downregulated DEGs Supplementary Figure 1A). Further expression analysis revealed that compared to the DMSO-treated group, the *SHCBP1* gene was downregulated in the 50 μM Rh7-treated group (Supplementary Figure 1B). The outcomes suggest that the downregulation of *SHCBP1* may be part of the molecular mechanisms through which Rh7 impacts GC cells.

### 3.5 Ginsenoside Rh7 inhibits the proliferation, migration, and invasion of GC cells

**Figure 4:**
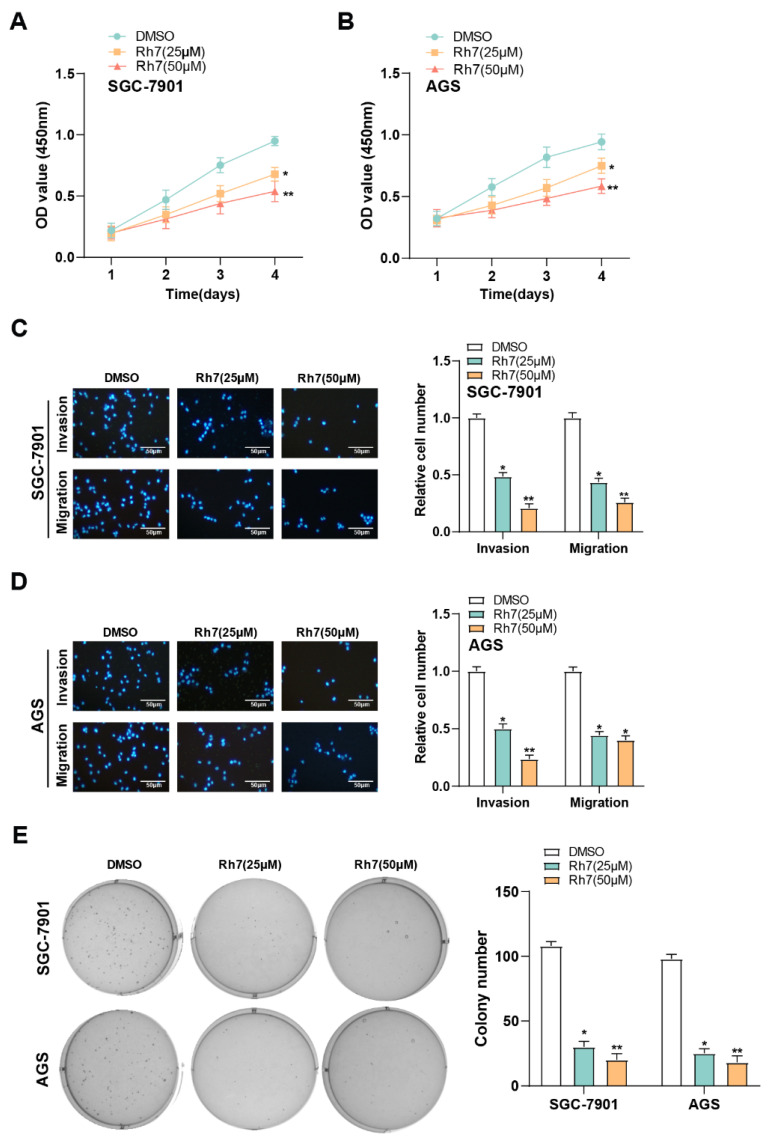
** Effect of ginsenoside Rh7 on the proliferation, migration, and invasion of GC cells.** (A and B) CCK-8 assay results showed the effect of 25 μM and 50 μM ginsenoside Rh7 on the proliferation of SGC-7901 and AGS cells. (C and D) Transwell assay images demonstrating the impact of 25 μM and 50 μM ginsenoside Rh7 on the migration and invasion of SGC-7901 and AGS cells, with corresponding bar graphs for quantification. Scale bar: 50 μm. (E) Clonogenic assay showing the effect of 25 μM and 50 μM ginsenoside Rh7 on colony formation in SGC-7901 and AGS cells. GC, gastric cancer; CCK-8, cell counting kit-8; DMSO: Dimethyl sulfoxide. *P< 0.05 or **P<0.01 vs. DMSO group.

The CCK-8 assay was used to evaluate the effect of ginsenoside Rh7 on GC cell proliferation. Treatment with 25 μM and 50 μM ginsenoside Rh7 resulted in a decrease in AGS and SGC-7901 cell proliferation rates (Figures 4A and 4B). Additionally, Transwell assays assessed the result of ginsenoside Rh7 on the capacity of GC cell lines to invade and migrate. Following ginsenoside Rh7 treatment, the number of invading and migrating SGC-7901 and AGS cells was notably decreased (Figures 4C and 4D). Clonogenic assays further demonstrated a notable decline in the number of colonies formed by SGC-7901 and AGS cells under the influence of ginsenoside Rh7 (Figure 4E). These outcomes collectively imply that ginsenoside Rh7 effectively inhibits GC cell invasion, migration, and proliferation.

### 3.6 Ginsenoside Rh7 decreases SHCBP1 expression in GC cell lines

**Figure 5:**
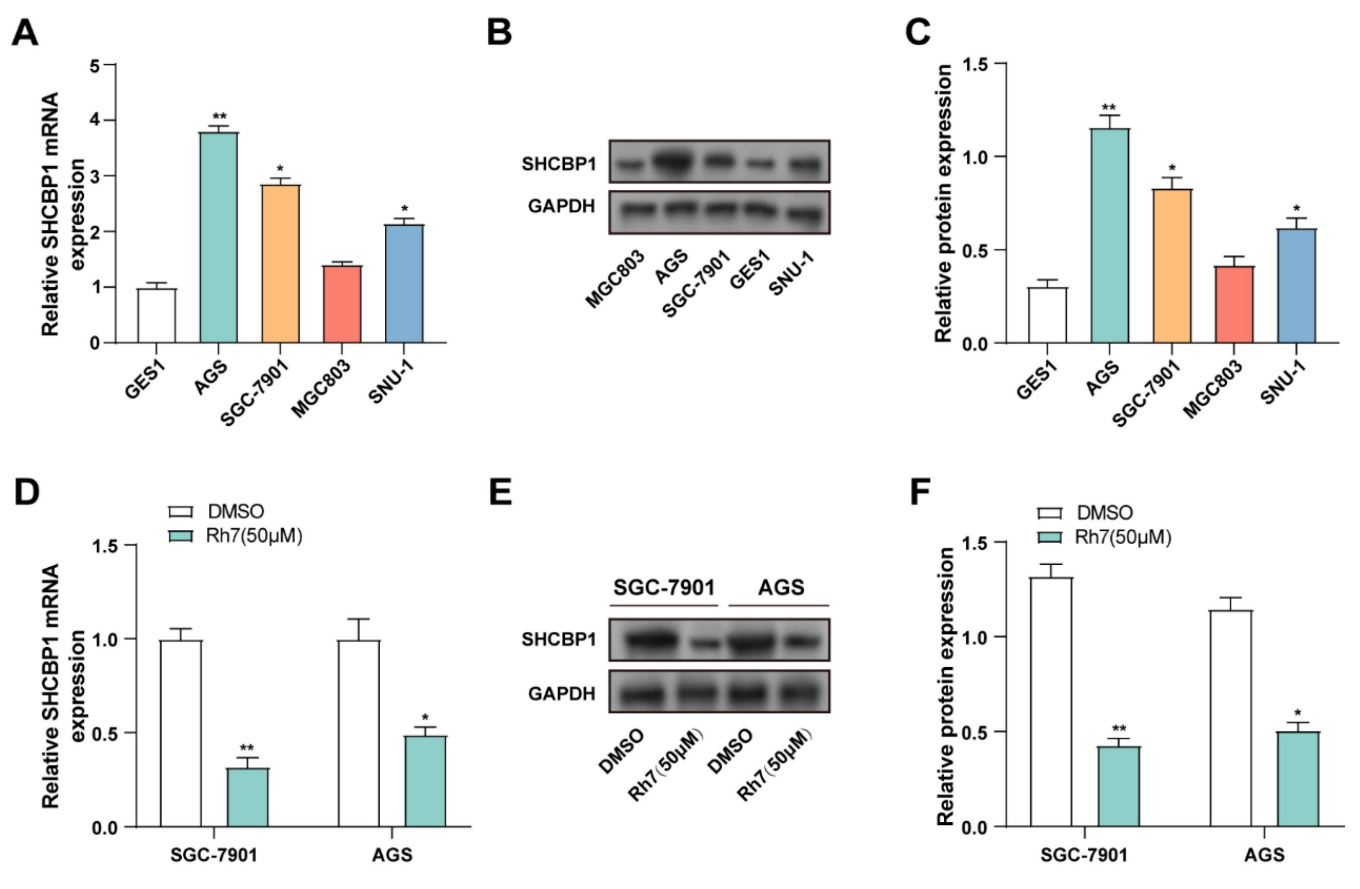
** Effect of ginsenoside Rh7 on *SHCBP1* expression in GC cell lines.** (A) *SHCBP1* expression was evaluated in normal human gastric epithelial cells (GES1) and GC cell lines (AGS, SGC-7901, MGC803, and SNU-1) by qRT-PCR. *P< 0.05 or **P<0.01 vs. GES1 group. (B and C) WB analysis of *SHCBP1* expression in GES1 and AGS, SGC-7901, MGC803, and SNU-1. The protein levels of *SHCBP1* were quantified by bar graphs. *P< 0.05 or **P<0.01 vs. GES1 group. (D) The effect of 50 μM ginsenoside Rh7 treatment on *SHCBP1* expression in AGS and SGC-7901 cells was measured by qRT-PCR. (E and F) WB analysis of *SHCBP1* expression following 50 μM ginsenoside Rh7 treatment in AGS and SGC-7901 cells. GC, gastric cancer; qRT-PCR, quantitative real-time polymerase chain reaction; WB, Western blot; DMSO: Dimethyl sulfoxide. *P< 0.05 or **P<0.01 vs. DMSO group.

*SHCBP1* expression was assessed in GES1, AGS, MGC803, SGC-7901, and SNU-1) using qRT-PCR and WB. Compared to GES1 cells, *SHCBP1* was significantly upregulated in AGS, SGC-7901, and MGC803 cells. Therefore, AGS and SGC-7901 cell lines, which exhibited the highest *SHCBP1* levels, were selected for further analysis (Figures 5A-5C). The effect of ginsenoside 50 μM Rh7 on *SHCBP1* expression was evaluated in GC cell lines using qRT-PCR and WB. *SHCBP1* levels significantly decreased after Rh7 treatment in comparison to the control group (Figures 5D-5F). According to these findings, ginsenoside Rh7 effectively suppresses *SHCBP1* expression in GC cells.

### 3.7 Ginsenoside Rh7 inhibits β-catenin nuclear translocation in GC cells by inhibiting SHCBP1-β-catenin interaction

**Figure 6:**
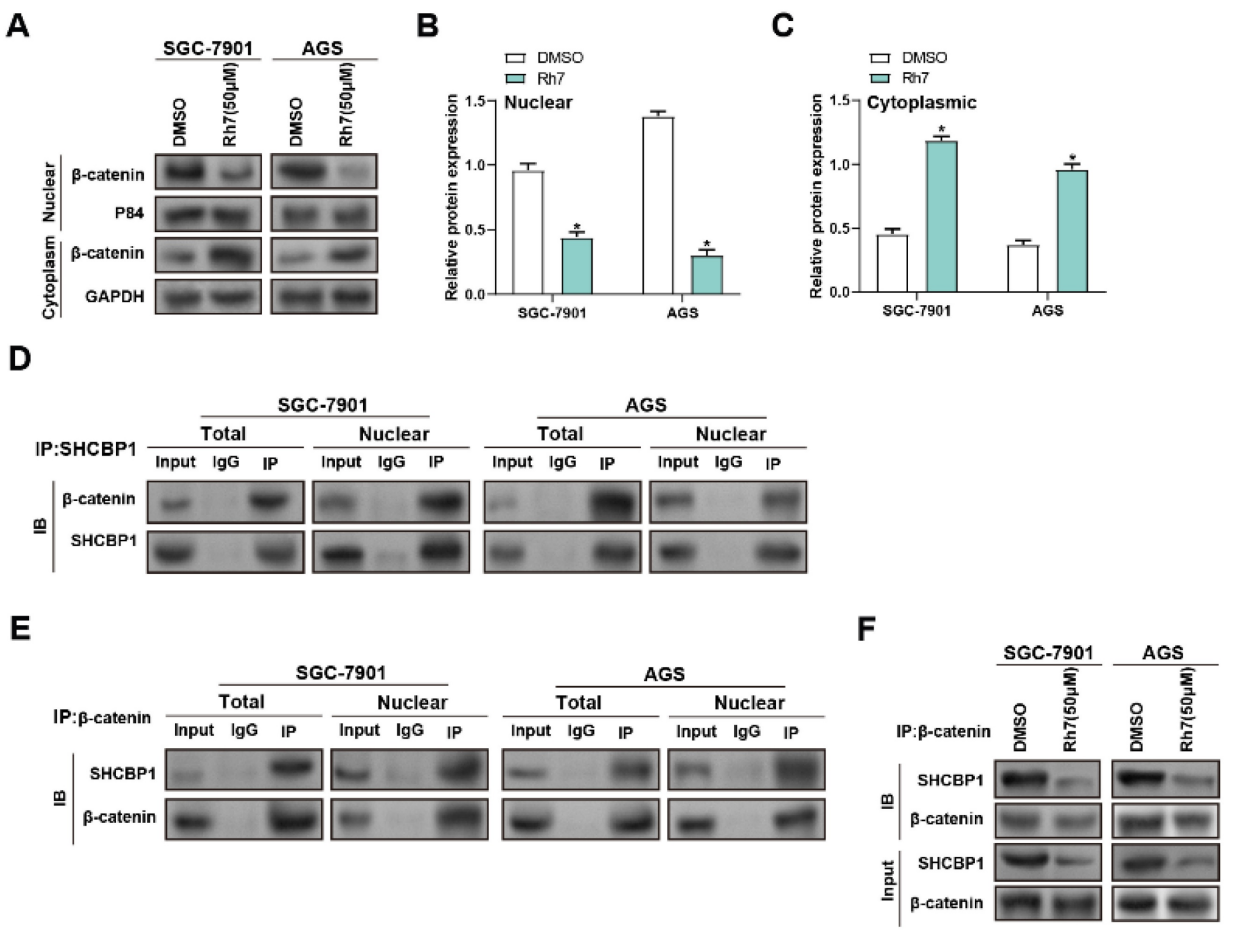
** Modulation of SHCBP1-β-catenin interaction and β-catenin nuclear translocation by ginsenoside Rh7 in GC cells.** (A-C) WB analysis of β-catenin levels in nuclear (nuc) and cytoplasmic (cyto) fractions of SGC-7901 and AGS cells after treatment with DMSO or 50 μM Rh7. P84 and GAPDH were used as nuclear and cytoplasmic markers, respectively, to confirm fractionation. (D and E) IP assays assessed the interaction between SHCBP1 and β-catenin in GC cells with 50 μM ginsenoside Rh7. (F) IP assays assessed of total SHCBP1 and β-catenin protein levels in SGC-7901 and AGS cells treated with DMSO or Rh7. GC, gastric cancer; WB, Western blot; IP: Immunoprecipitation.

β-catenin is a cell membrane adhesion complex component that promotes tight junctions between adjacent cells(20. To research the impact of Rh7 treatment on the interaction between SHCBP1 and β-catenin, WB and IP assays were performed. Rh7 treatment led to a notable reduction in nuclear β-catenin levels, accompanied by an increase in cytoplasmic β-catenin expression, in both SGC-7901 and AGS cells compared to DMSO-treated controls. GAPDH was used as a marker for the cytoplasm, and P84 was used as a marker for the nucleus to verify the quality and accuracy of protein isolation (Figure 6A-6C). IP assays were then conducted to examine the relationship between SHCBP1 and β-catenin. Under DMSO treatment, β-catenin exhibited a strong interaction with SHCBP1 in both the total and nuclear protein fractions, with higher expression observed in the nucleus (Figures 6D and 6E). WB analysis of total protein lysates proved that Rh7 treatment reduced the levels of SHCBP1 and β-catenin in SGC-7901 and AGS cells compared to the control group for DMSO (Figure 6F). These outcomes reveal that Rh7 facilitates the nuclear translocation of β-catenin through interaction with *SHCBP1*.

### 3.8 Ginsenoside Rh7 modulates EMT in GC cells via SHCBP1-mediated β-catenin translocation

**Figure 7:**
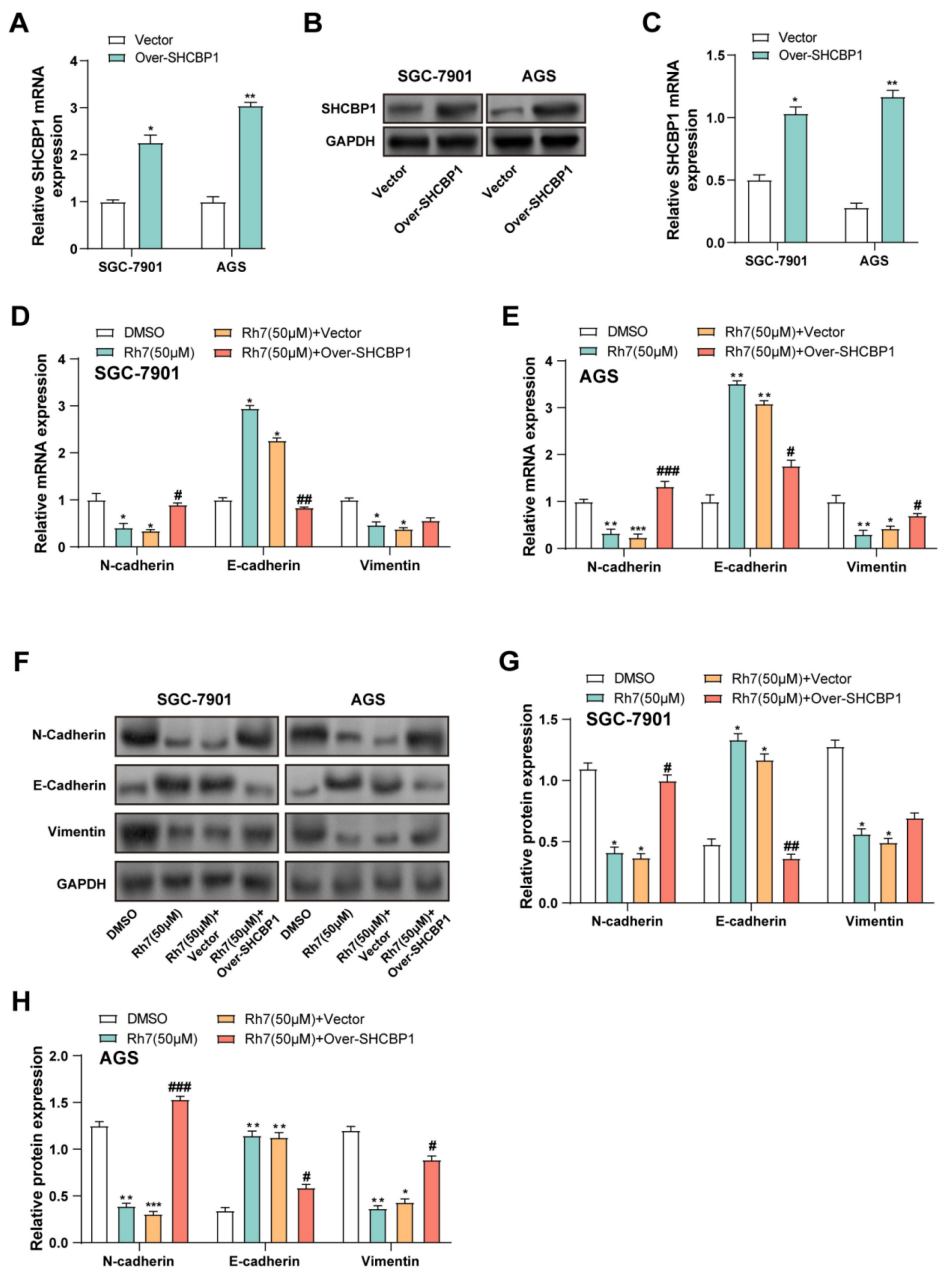
** Regulation of EMT in GC cells by ginsenoside Rh7 through *SHCBP1*-mediated β-catenin translocation.** (A-C) Transfection efficiency of *SHCBP1* overexpression plasmids in SGC-7901 and AGS cells, evaluated by qRT-PCR (A) and WB (B and C). *P< 0.05 or **P<0.01 vs. vector group. (D and E) Effects of Rh7 treatment and *SHCBP1* overexpression on the expression of EMT markers (*N-cadherin*, *E-cadherin*, and *Vimentin*) in GC cells, assessed by qRT-PCR. (F-H) WB analysis of the expression of EMT-related factors (N-cadherin, E-cadherin, and Vimentin) in GC cells following Rh7 treatment and *SHCBP1* overexpression, quantified using bar graphs. GC, gastric cancer; qRT-PCR, quantitative real-time polymerase chain reaction; WB, Western blot; EMT, epithelial-mesenchymal transition; DMSO: Dimethyl sulfoxide. *P< 0.05 or **P<0.01 or ***P<0.001 vs. DMSO group. ^#^*P*< 0.05 or ^##^*P*<0.01 or ^###^*P*<0.001 vs. Rh7 (50μM) + vector group.

Given that ginsenoside Rh7 can reduce the expression level of *SHCBP1* in GC cells, we aimed to explore whether overexpression of *SHCBP1* could reverse the changes in cell phenotype and related protein expression caused by Rh7. Therefore, we selected two cell lines, AGS and SGC-7901, for *SHCBP1* overexpression experiments. qRT-PCR and WB analysis confirmed the successful overexpression of *SHCBP1* in cells treated with the *SHCBP1* overexpression plasmid in SGC-7901 and AGS. Both the mRNA and protein expression of* SHCBP1* were markedly elevated in the overexpression group in contrast to the vector control group (Figures 7A-7C). qPCR analysis showed that the mRNA expression levels of *N-cadherin*, *E-cadherin*, and *Vimentin* changed in SGC-7901 and AGS cells after different treatments. Compared with the DMSO control group, Rh7 (50 μM) reduced the expression of *N-cadherin* and *Vimentin*, while increasing the expression of *E-cadherin*. When cells were overexpressed with *SHCBP1* and treated with Rh7, *N-cadherin* expression rebounded, while *E-cadherin* expression decreased. In SGC-7901 cells, *SHCBP1* overexpression had little effect on the changes in *Vimentin* caused by Rh7, but the effect was more significant in AGS cells (Figures 7D and 7E). These findings are consistent with the results of Supplementary Figure 2A-2C, in which *SHCBP1* overexpression increased the expression of *N-cadherin* and *Vimentin* and decreased the expression of *E-cadherin* in AGS cells. At the same time, the group overexpressing *SHCBP1* and treated with Rh7 showed a decrease in the expression of *N-cadherin* and *Vimentin* and a rebound in the expression of *E-cadherin*. WB analysis further confirmed these results and was consistent with the mRNA expression results (Figures 7F-7H and Supplementary Figures 2D and 2E). These results suggest that Rh7 affects the EMT and proliferation behaviors of GC cells by regulating *SHCBP1*-mediated β-catenin activity, and overexpression of *SHCBP1* may somewhat offset these effects of Rh7.

### 3.9 Ginsenoside Rh7 modulates GC cell invasion and migration through SHCBP1

**Figure 8:**
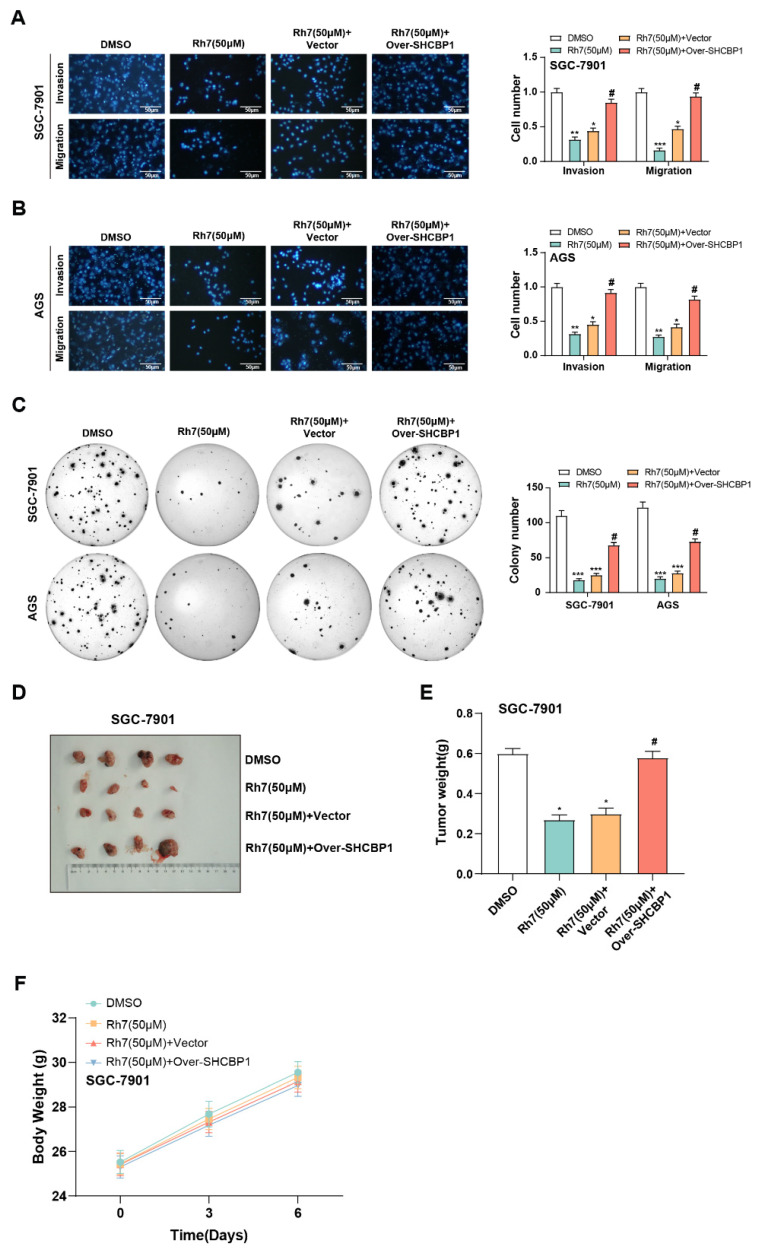
** Effects of ginsenoside Rh7 and *SHCBP1* overexpression on GC cell migration, invasion, proliferation, and tumor growth.** (A and B) Transwell assays assessing the effects of ginsenoside Rh7 treatment and *SHCBP1* overexpression on the migration and invasion of GC cells. Scale bar: 50 μm. (C) Clonogenic assays measuring the impact of *SHCBP1* overexpression on GC cell proliferation in the presence of Rh7 treatment. The x-axis indicates the treatment conditions, and the y-axis shows the number of colonies formed by GC cells. (D-F) *In vivo* tumor model studies evaluating the effect of Rh7 treatment and *SHCBP1* overexpression on tumor size and weight. GC, gastric cancer; DMSO: Dimethyl sulfoxide. *P< 0.05 or **P<0.01 or ***P<0.001 vs. DMSO group.^ #^*P*< 0.05 vs. Rh7 (50μM) + vector group.

In our reactive experiments, we investigated the effects of Rh7 and SHCBP1 overexpression on GC cell growth. Transwell assay results showed that Rh7 treatment significantly inhibited the migration and invasion of GC cells. However, when *SHCBP1* was overexpressed in cells, the inhibitory effect of Rh7 on these processes was attenuated (Figures 8A and 8B). In addition, clone formation assays further confirmed that *SHCBP1* overexpression could alleviate the inhibitory effect of Rh7 on the proliferation of GC cells (Figure 8C). Transwell assays in Supplementary Figure 2F further showed that AGS cells overexpressing *SHCBP1* had enhanced migration and invasion abilities. However, when these cells were simultaneously treated with Rh7, their migration and invasion abilities were reduced. This view was also supported by clone formation assay results in Supplementary Figure 2G, showing that overexpression of *SHCBP1* increased the number of clones formed by AGS cells. However, in the group where *SHCBP1* was overexpressed and treated with Rh7, the number of clones formed was reduced. In addition, the results of *in vivo* studies using mouse tumor models were consistent with those of *in vitro* experiments. The tumor volume of mice in the Rh7-treated group was smaller, while the Rh7 + *SHCBP1* overexpression group reversed the effect of Rh7 treatment (Figures 8D and 8E). The body weight of mice in all treatment groups increased over time, but the growth trend was similar, indicating that Rh7 treatment did not significantly affect the overall health or body weight of mice at this dose (Figure 8F). These findings together highlight the potential of Rh7 to exert its antitumor effects through the *SHCBP1*-β-catenin axis.

## 4. Discussion

This study utilized bioinformatics approaches, including WGCNA, differential expression analysis, and PPI network analysis, to identify two overlapping genes, *MELK* and *SHCBP1*, closely associated with GC. The study by Su P *et al.* revealed that *MELK* overexpression in GC is associated with chemoresistance and M2 macrophage polarization^21^. These results imply that *MELK* promotes chemoresistance in the tumor microenvironment by the CSF-1/JAK2/STAT3 pathway, highlighting its potential as a novel therapeutic target and prognostic marker. Additionally, Jiang F *et al.* performed an in-depth investigation of the expression of *SHCBP1* across 33 cancer types, examining its associations with prognosis, immune microenvironment, tumor mutational burden, and microsatellite instability^22^. Experimental results further revealed high *SHCBP1* expression in gastrointestinal cancers, underscoring its potential value in tumor immunotherapy. Given the great research potential of* SHCBP1* in GC, this study focused on revealing its molecular mechanism and its role in GC progression.

Ginsenosides are compounds extracted from the natural plant ginseng, which have important research value in many fields such as anti-tumor, anti-inflammatory, antioxidant and anti-aging treatment^23^-^25^. Yao W and Guan Y emphasized that ginsenoside Rg3 slowed the development of colorectal cancer by inhibiting the nuclear translocation of C/EBP β and its interaction with NF-kB, thereby inhibiting the inflammatory pathway^26^. Shah MA *et al.* analyzed the anti-tumor effects of Rb1, Rc, Rd, Rg3 and Rh2^27^. These compounds produce anti-tumor activity by triggering apoptosis, controlling the cell cycle, and regulating multiple signaling pathways like the NF-kB pathway, the p53 pathway, and the inflammatory pathway. The review by Yang Y *et al.* also summarized the effects of 20 ginsenoside subtypes in inhibiting tumor cell proliferation, promoting cell apoptosis, inhibiting invasion and migration^28^. Ginsenoside Rh7 is a rare protopanaxatriol (PPT) type saponin. Compared with other ginsenosides (such as Rg3, Rd, etc.), Rh7 has unique chemical properties and higher pharmacological activity due to the small number of glycosylation modifications. At present, there are relatively few studies on ginsenoside Rh7, but existing studies have shown that it is involved in tumor proliferation inhibition, anti-inflammation, and immunomodulation. This study showed that ginsenoside Rh7 exhibited significant anti-tumor effects on GC cells through cell experiments. Rh7 cell viability and suppressed cell proliferation show dose dependency, especially at 50 μM and 100 μM doses. In addition, Rh7 inhibited GC cell invasion, migration, and colony formation, highlighting its potential as a therapeutic agent against GC progression.

*SHCBP1* is a critical signaling molecule, and several studies have explored its therapeutic significance in various types of cancer. Research conducted by Shi W *et al.* showed that HER2 promotes cell mitosis by activating the HER2-*SHCBP1*-PLK1 axis, thereby influencing the development of GC and the sensitivity to trastuzumab^29^. High expression of *SHCBP1* was associated with poor response to trastuzumab treatment in patients. Research by Ren C *et al.* found that *SHCBP1* is elevated in esophageal tissues and is linked to clinical features in individuals with esophageal squamous cell carcinoma (ESCC)^30^. Loss of *SHCBP1* inhibited ESCC cell proliferation and migration through the TGF-β pathway and suppressed tumor growth in mice. These findings suggest a potential link between *SHCBP1* regulation and cancer progression, prompting further investigation into the role of *SHCBP1* in the therapeutic effects of ginsenoside Rh7 on GC. In this study, transcriptome sequencing was employed to analyze gene expression changes in GC cells exposed to ginsenoside Rh7. The findings showed a significant increase of the *SHCBP1* gene in the Rh7-treated group. Furthermore, ginsenoside Rh7 markedly reduced *SHCBP1* expression in GC cell lines, suggesting that Rh7 may exert its anticancer effects by inhibiting *SHCBP1* expression.

β-catenin, a crucial part of the Wnt/β-catenin cascade, participates in cell proliferation, migration, differentiation, and tumorigenesis^31^. Tumor growth and metastasis in several malignancies are tightly linked to aberrant β-catenin accumulation and nuclear translocation^32^, ^33^. Previous studies have found that EGFR activation promotes the release of *SHCBP1*, which in turn enhances the transcriptional activity of β-catenin, driving stemness and malignant progression in NSCLC cells. A recent investigation by Ding *et al.* disclosed that the deubiquitinase USP49 stabilizes *SHCBP1* in esophagogastric junction adenocarcinoma, promoting β-catenin nuclear translocation^34^. This, in turn, enhances the transcriptional expression of glutathione peroxidase 4, inhibiting ferroptosis and cell proliferation. In the present study, we observed that ginsenoside Rh7 treatment significantly reduced the nuclear translocation of β-catenin in GC cells and decreased the expression of *SHCBP1* by WB and IP experiments. These results suggest that Rh7 may exert its anti-tumor effect by regulating *SHCBP1*-mediated β-catenin activity. Specifically, Rh7 treatment significantly decreased nuclear β-catenin levels in SGC-7901 and AGS cells, while increasing cytoplasmic β-catenin expression. This suggests that Rh7 may inhibit the activation of the Wnt/β-catenin signaling pathway by inhibiting the interaction between *SHCBP1* and β-catenin and preventing β-catenin from translocating from the cytoplasm to the nucleus. This finding is consistent with previous studies, in which EGF-induced *SHCBP1* nuclear translocation promoted the interaction between β-catenin and CBP, increasing β-catenin acetylation and activating stemness-related gene transcription^35^. Therefore, the role of Rh7 may be through interfering with the formation of *SHCBP1*-β-catenin complex, affecting the nuclear translocation of β-catenin and the activation of Wnt signaling pathway, which provides a potential molecular mechanism for Rh7 as an anti-tumor agent.

EMT causes the disappearance of epithelial traits and the gain of mesenchymal traits by epithelial cells, promoting tumor malignancy and dissemination^36^. Baj J *et al.* discussed that Helicobacter pylori and its virulence factors (such as CagA, YAP pathway, and MMP-7) promote tumor progression and cancer stem cell properties in GC through EMT^37^. The main characteristics of EMT include increases in N-cadherin and decreases in E-cadherin, which propel EMT^38^. The mesenchymal marker vimentin is strongly linked with tumor migration and invasion. Zhang Y *et al.* found that VEGF and EMT markers in gastric cancer tissues were strongly correlated with the stage, metastasis, and depth of tumor invasion, highlighting their role in GC progression^39^. EMT is closely related to β-catenin signaling, and abnormal nuclear translocation of β-catenin drives EMT initiation through transcriptional regulation of downstream targets, thereby further enhancing tumor invasion and metastasis^40^. In this study, Rh7 regulated GC cell EMT and invasion through *SHCBP1*-mediated β-catenin signaling. Rh7 treatment downregulated mesenchymal markers (Vimentin and N-cadherin) and elevated E-cadherin, thereby inhibiting EMT. However, *SHCBP1* overexpression reversed these effects and partially restored the expression of EMT markers. Functionally, Rh7 reduced the invasion, migration, and proliferation of GC cells. *In vivo*, Rh7 suppressed tumor size and weight, while *SHCBP1* overexpression attenuated these effects. These findings suggest that Rh7 exerts its antitumor effects by targeting the *SHCBP1*-β-catenin pathway in GC cells.

Although our results reveal the inhibitory effects of Rh7 on gastric cancer cell proliferation, migration, and invasion in both *in vitro* and *in vivo* models, some limitations still need to be addressed in future studies. First, our study has not directly evaluated the effect of Rh7 on the expression levels of key downstream target genes of the Wnt/β-catenin pathway. This requires detailed analysis by methods such as qRT-PCR, WB, or RNA sequencing to understand the mechanism of action of Rh7 fully. Second, although we observed that Rh7 may exert its effect by affecting the interaction between *SHCBP1* and β-catenin, the specific molecular mechanism of this effect has not been fully elucidated. Future studies need to explore further how Rh7 precisely regulates this interaction and how these changes affect downstream signaling pathways and cell behavior. In addition, our functional validation experiments mainly rely on overexpression and interference RNA technology to study the role of *SHCBP1*, and these results need to be supported by more functional validation experiments to support the necessity of *SHCBP1* in the action of Rh7. Finally, our studies were mainly conducted *in vitro* and animal models, and their clinical relevance and application potential need to be further verified by clinical sample analysis and potential clinical trials.

## 5. Conclusion

In conclusion, our outcomes indicate that ginsenoside Rh7 exerts antitumor impacts on GC cells by preventing invasion, migration, and proliferation. This research emphasizes the function of *SHCBP1* in regulating these effects, with Rh7 inhibiting *SHCBP1* expression and promoting β-catenin nuclear translocation. Mechanistically, Rh7 disrupts the connection between β-catenin and *SHCBP1*, reduces β-catenin nuclear translocation, and regulates EMT markers, thereby inhibiting EMT and related tumorigenic behaviors. Further* in vivo* experiments confirmed the antitumor effects of Rh7, which were partially reversed by* SHCBP1* overexpression. These findings collectively suggest that Rh7 exerts its antitumor effects via the SHCBP1-β-catenin axis, offering novel viewpoints on the biological processes underlying the development of GC.

## Supplementary Material

Supplementary figures.
