# Supplementary material for: Ginsenoside Rh7 affects β-catenin nuclear translocation by inhibiting SHCBP1 expression, thereby inhibiting epithelial-mesenchymal transition in gastric cancer cells
Source: Int J Med Sci. 2025 Jun 23;22(12):3053–69. doi: 10.7150/ijms.112622 (PMC12244034; doi:10.7150/ijms.112622)
Supplement: Supplementary file 1 — Supplementary figures. [file ijmsv22p3053s1.pdf]

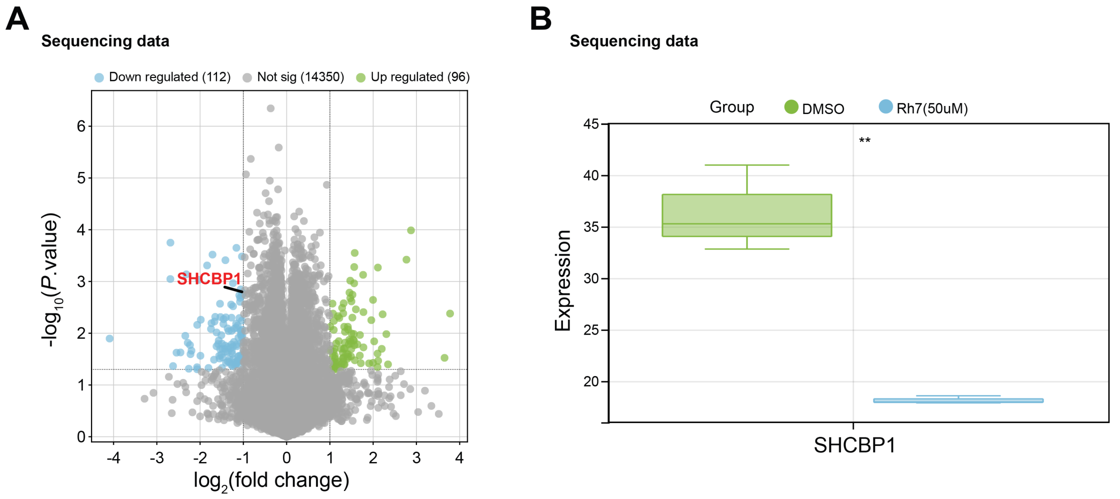

Supplementary Figure 1. Transcriptomic analysis of *SHCBP1* expression in SGC-7901 cells after treatment with ginsenoside Rh7.

(A) Volcano plot illustrating the differential expression of genes in gastric cancer cells following treatment with 50 μM ginsenoside Rh7. Upregulated genes are indicated in blue, and downregulated genes are in green. The x-axis represents $\log_2$ fold changes, and the y-axis represents the $-\log_{10}$ p-value. (B) Expression levels of *SHCBP1* in gastric cancer cells treated with 50 μM ginsenoside Rh7, compared to the DMSO-treated control group, as determined by RNA-sequencing analysis. The x-axis represents treatment conditions, and the y-axis represents the normalized expression levels of *SHCBP1*. DMSO: Dimethyl sulfoxide; RNA: Ribonucleic acid. $^{**}P<0.01$ vs. DMSO group.

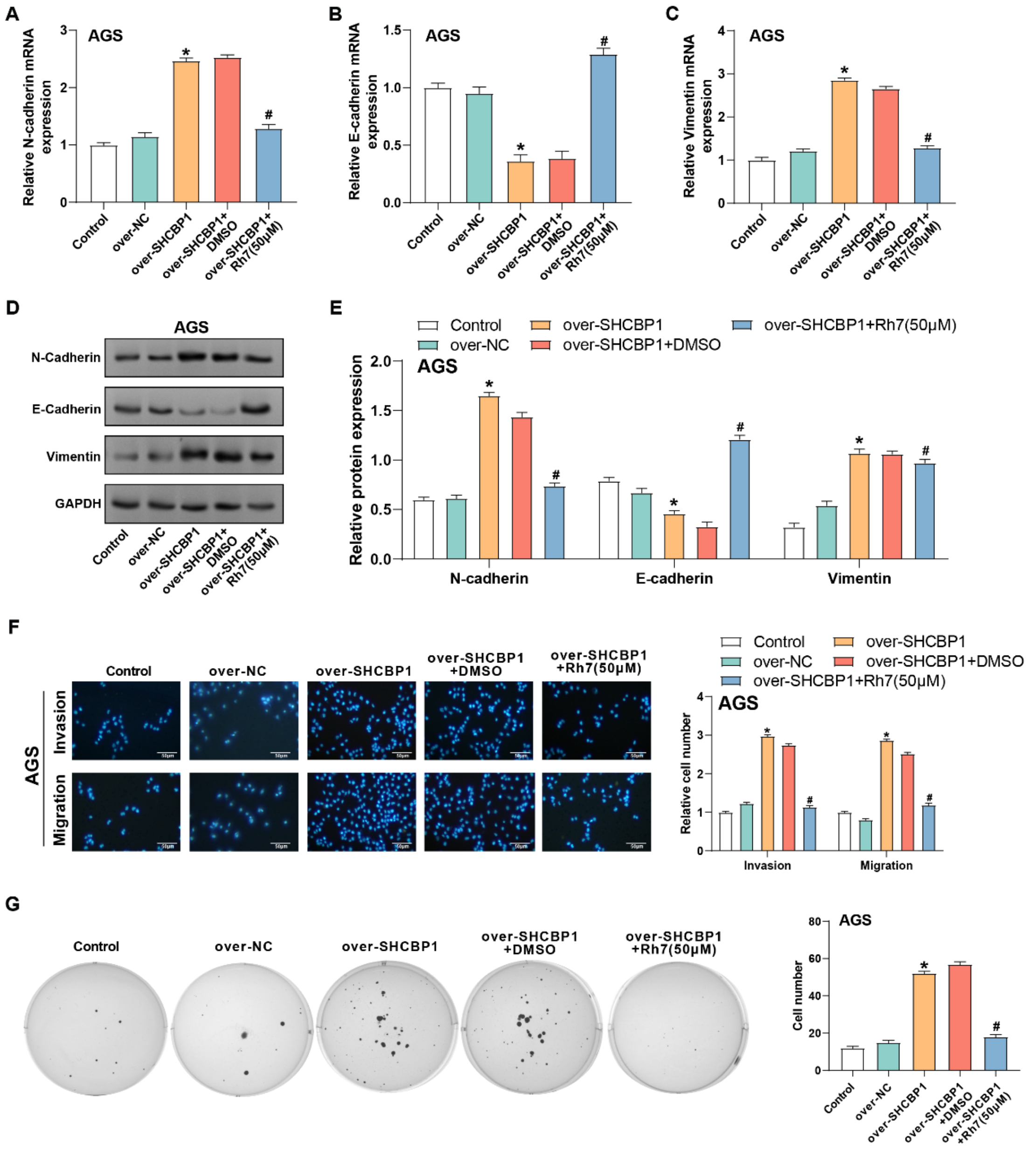

Supplementary Figure 2. Effects of ginsenoside Rh7 on *SHCBP1* expression and Wnt/β-catenin signaling pathway in SGC-7901 and AGS cells

(A-C) The effects of *SHCBP1* overexpression and Rh7 treatment on the expression of EMT markers (*N-cadherin*, *E-cadherin*, and *Vimentin*) in GC cells were evaluated by qRT-PCR. (D and E) Western blot analysis of the expression of EMT-related factors (N-cadherin, E-cadherin, and vimentin) in GC cells after *SHCBP1* overexpression and Rh7 treatment, quantified using bar graphs. (F) Transwell assay to evaluate the effects of *SHCBP1* overexpression and ginsenoside Rh7 treatment on GC cell migration and invasion. Scale bar: 50 μm. (G) Clonogenic assay measuring GC cell proliferation in

the presence of *SHCBP1* overexpression and Rh7 treatment. The x-axis represents the treatment conditions, and the y-axis represents the number of colonies formed by GC cells. GC, gastric cancer. *P< 0.05 vs. Control group. #P< 0.05 vs. over-*SHCBP1*.
